# Enhanced Bézier curve-based trajectory planning for high-altitude autonomous trucks

**DOI:** 10.1371/journal.pone.0333685

**Published:** 2025-09-26

**Authors:** Du Chigan, Jianbei Liu, Yang Zhao, Jianyou Zhao

**Affiliations:** 1 School of Transportation Engineering, Chang’an University, Xi’an, China; 2 CCCC First Highway Consultants Co., Ltd., Xi’an, China; 3 Capital Construction Department, Chang’an University, Xi’an, China; 4 School of Automobile, Chang’an University, Xi’an, China; Southwest Jiaotong University, CHINA

## Abstract

Highway freight transport is the backbone of Tibet’s logistics network, accounting for 76.4% of regional freight movement (Tibet Bureau of Statistics, 2024). Challenging alpine road conditions—characterized by steep grades, sharp curves, and narrow lanes—combine with the substantial dimensions of heavy trucks to create significant operational difficulties. Autonomous truck development offers a potential solution; however, their trajectory planning algorithms exhibit limitations in high-altitude environments. To address these challenges, we propose a novel trajectory planning method using quartic Bézier curves. These 4th-order parametric curves provide G² continuity. Our approach integrates speed profiles into a three-dimensional curve representation and employs a two-phase optimization process to ensure safety and efficiency. Simulation results demonstrate the method’s effectiveness in maintaining truck stability while enabling responsive maneuvering under Tibet’s demanding road conditions.

## 1. Introduction

Highway freight transportation serves as the lifeline of Tibet’s logistics sector, comprising 76.4% of the region’s total freight movement and underscoring its irreplaceable role in sustaining cross-regional connectivity (Table 1) [1]. While Tibet’s highway network spans approximately 120,000 kilometers, a stark infrastructure disparity exists: Only 1,196 kilometers (0.8%) meet first-grade standards, with the overwhelming majority (99.2%) classified as second-grade or lower [2]. This deficit is particularly evident along Tibet’s five main access routes, which suffer from substandard road classifications, irregular alignments, narrow lanes, vulnerability to natural disasters, and frequent single-lane maintenance.

**Table 1:** Traffic volume on the Qinghai-Tibet highway. The table describes the traffic volume and proportion of different vehicle types in the Wudaoliang section for 2024.

Vehicle type	Small truck	Medium truck	Large truck	Extra-large truck	Small passenger	Large passenger	Motorcycle
Natural number	57920	9102	22328	261079	140804	3756	3112
Percentage (%)	11.63	1.83	4.48	52.41	28.27	0.75	0.62

**Fig 1:**
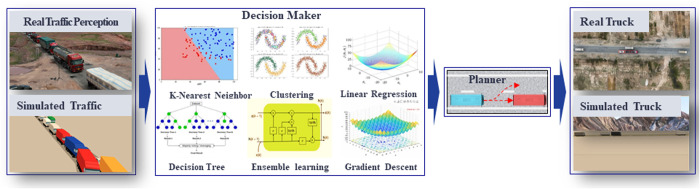
Truck collision avoidance trajectory planning system. The system perceives real-time traffic conditions, simulates scenarios via modeling, formulates decisions through intelligent algorithms, completes trajectory planning, and finally executes lane changes and trajectory tracking via the controller.

Intelligent transformation in the automotive sector has significantly advanced commercial vehicle technologies, particularly in sensor fusion, algorithmic decision-making, and vehicle control architectures. Global pilot programs deploying autonomous trucks in real-world conditions have generated valuable empirical data. This data has enabled substantial improvements in obstacle avoidance and trajectory planning methodologies [3–6]. Contemporary autonomous trucks (Fig 1) incorporate three core functional layers. Sophisticated sensor fusion algorithms enhance their multi-modal sensor suites (LiDAR, visual odometry (VO), GNSS, and HD maps). These suites enable centimeter-level real-time environmental perception and reliable dynamic object tracking [7–9]. Building on this perception, hierarchical decision-making frameworks process sensory inputs. They generate context-aware maneuvers that optimally balance safety margins, traffic regulations, and operational efficiency in real-time. Advanced drive-by-wire systems execute these decisions. These systems provide decoupled control of steering, braking, and propulsion. This enables precise trajectory following (sub-decimeter accuracy) while maintaining vehicle stability within defined safety margins [10–12].

Despite these advancements, developing adaptive trajectory optimization algorithms that generalize across operational domains remains challenging. Specifically, decision-making for challenging scenarios—like low-friction surfaces, mixed traffic, or geometrically constrained roads—requires breakthroughs. Key areas include reinforcement learning, probabilistic risk assessment, and real-time computational efficiency. These gaps necessitate hybrid architectures that combine rule-based systems with machine learning. Such architectures ensure robust performance under environmental uncertainty.

The structure of this paper is as follows. Section 2.1 formulates the core research problem and establishes the methodological framework for its solution. Section 2.2 develops a comprehensive trajectory and velocity planning algorithm for the collision avoidance phase, complemented by Section 2.3 which presents the corresponding planning methodology for the lane-recovery phase. Section 3 systematically validates the proposed approach through numerical simulations and provides an in-depth analysis of the results. The paper concludes with Section 4, which summarizes key findings and discusses their implications.

The field of trajectory planning has achieved remarkable progress through innovative methodologies addressing diverse vehicular applications. Tsai’s algebraic general trajectory formulation (GTT) [13] revolutionized semi-trailer path planning by incorporating dimensional and turning constraints to ensure comprehensive vehicle coverage. Ozana’s six-stage optimization framework [14] established new standards by harmonizing global and local planning techniques. Subsequent developments have specialized these approaches, including Wei’s fifth-degree polynomial CAV model [15] and Yang’s truck-specific lane-changing adaptation [16]. The field has further evolved through Li Bai’s mixed-integer kinematic solution [17], Li QW’s analytical platooning framework [18], and Zhang’s sophisticated 7-DOF articulated vehicle control [19], demonstrating both theoretical depth and practical applicability.

Emergency obstacle avoidance research has similarly advanced through scenario-specific innovations [20,21]. Shi’s rollover-preventive algorithm [22] and Li’s mining truck optimization [23] represent critical safety-focused developments. Zhan’s TOPSIS-based curved road solution [24] and Zhou’s platoon coordination model [25] address complex operational environments, while Shi Yue’s integrated maneuver system [26] and Muralidhara’s narrow-road articulation method [27] provide comprehensive control frameworks. Recent breakthroughs include Li Ning’s four-wheel steering emergency system [28], Zhang’s Bézier-based avoidance [29], Tian’s lane-safety optimization [30], and Chen’s segmented 3D rescue vehicle approach [31], collectively expanding the field’s technical boundaries.

Existing trajectory planning methods fall into five main categories: Bézier curve-based, polynomial-based, model predictive control (MPC), sampling-based optimization, and reinforcement learning. Table 2 summarizes the strengths and limitations of each. Bézier curves are particularly valuable for trajectory planning. They offer curvature continuity, smoothness, ease of shape control, and convex hull safety properties.

**Table 2:** Comparison of Trajectory Planning Methods for heavy-duty truck operations at high-altitude. The table compares the advantages and limitations of Bézier curve planning, polynomial-based methods, model predictive control (MPC), sampling-based techniques, and reinforcement learning approaches.

Method	Advantages	Limitations
Bézier Curves	➢ Mathematically elegant and computationally efficient. ➢ Smooth paths suitable for truck dynamics. ➢ Easy to adjust via control points.	➢ Limited adaptability to complex terrains. ➢ Weak real-time obstacle avoidance. ➢ Insensitive to aerodynamic changes at high altitudes.
Polynomial Planning	➢ High-order continuity for precise control. ➢ Ideal for structured roads. ➢ Flexible parameterization.	➢ Prone to oscillations on unstructured terrain. ➢ High computational cost with increasing order. ➢ Slow response to dynamic environments.
Model Predictive Control	➢ Dynamic optimization for changing conditions. ➢ Incorporates truck dynamics and constraints. ➢ Good real-time performance.	➢ Computationally intensive. ➢ Model accuracy affected by thin air. ➢ Requires expert tuning.
Sampling-Based Methods	➢ Global planning for complex terrain. ➢ Handles high-dimensional spaces. ➢ Strong obstacle avoidance.	➢ Trajectories may lack smoothness. ➢ Poor real-time performance. ➢ High computational load.
Reinforcement Learning	➢ Adapts to environmental changes. ➢ Learns complex driving policies. ➢ Suitable for long-term tasks.	➢ Requires extensive training data. ➢ Generalization in high-altitude scenarios unproven. ➢ Unstable real-time decisions.

Current research primarily focuses on trajectory planning for trucks, often decoupling speed planning in real-time operations. This disconnect can produce dynamically infeasible trajectories. Trucks must then make abrupt adjustments to follow the path. Such compromises degrade ride comfort, introduce planning-execution delays, reduce efficiency, increase energy consumption, and risk loss of vehicle control—especially during high-speed travel on sharp curves. Although truck trajectory planning is evolving from single- to multi-scenario applications, research on coupled scenarios—integrating special road conditions, traffic dynamics, and vehicle constraints—remains insufficient.

The Tibetan operating environment presents unique challenges demanding specialized solutions. Trucks servicing this region combine substantial mass properties (elevated center of gravity, extended dimensions, and wide turning radii, Table 3) with demanding infrastructure constraints (low-grade narrow roads, poor alignment, frequent maintenance zones, and geological hazards). These conditions necessitate trajectory solutions with exceptional three-dimensional stability, stringent spatial precision, and optimized lane transition efficiency.

**Table 3:** Key parameters of main truck types on the Qinghai-Tibet highway. The table presents key parameters of four industry-standard vehicle models, including dimensions (L × W × H), maximum power output, gross vehicle mass, and minimum turning radius.

Types	Length (m)	Width (m)	Height (m)	Maximum power (kW)	Total traction mass (t)	Minimum turning radius (m)
1	15.12	2.53	3.53	426	40	7.69
2	17.15	2.65	3.99	164	18	6.75
3	15.42	2.50	3.71	338	39.61	5.4
4	20.00	2.55	3.97	418	25	11.5

Bézier curves offer particular advantages through their intrinsic mathematical properties: curvature continuity, parametric control via convex hull confinement, and smooth transitional characteristics. Compared to cubic Bézier curves, quartic Bézier curves can achieve continuity in position (G⁰), tangent direction (G¹), and curvature (G²) at junctions. Meanwhile, relative to quintic Bézier curves, quartic curves satisfy G² continuity while offering higher computational efficiency and avoiding overfitting. Despite their proven utility in conventional vehicle applications, existing research fails to address either the Tibetan highway context or the critical speed-trajectory coordination required for safe obstacle avoidance in these extreme conditions.

This study bridges this critical gap by developing a novel Bézier-based trajectory planning framework specifically designed for Tibetan highway obstacle scenarios. Under the premise of acquiring road obstacle and surrounding vehicle information through vision and radar systems [5,8,12], Our innovative approach establishes a direct functional mapping between spatial coordinates and velocity profiles through vertical axis parameterization, enabling simultaneous multi-objective optimization of safety, stability, and operational efficiency, the planned trajectory is accurately executed through precision driving assistance and actuation systems. The proposed method represents a significant advancement in addressing the unique challenges of heavy-duty trucks operations at high-altitude, while maintaining both mathematical elegance and computational practicality.

## 2. Methodology

### 2.1 Problem description and solution design

Trajectory planning for trucks navigating obstacle avoidance scenarios on Tibetan highways necessitates comprehensive integration of multi-source environmental data with the region’s unique roadway conditions, coupled with synchronized speed planning. This study employs three-dimensional Bézier curves as the mathematical foundation for trajectory generation, innovatively parameterizing speed as the vertical coordinate within the trajectory plane. This novel formulation establishes a real-time bijective mapping between velocity profiles and spatial positions, significantly enhancing vehicular maneuverability. The unified framework for speed-curvature co-design enables simultaneous optimization of three critical performance metrics: vehicle stability, operational efficiency, and dynamic handling.

**Fig 2:**
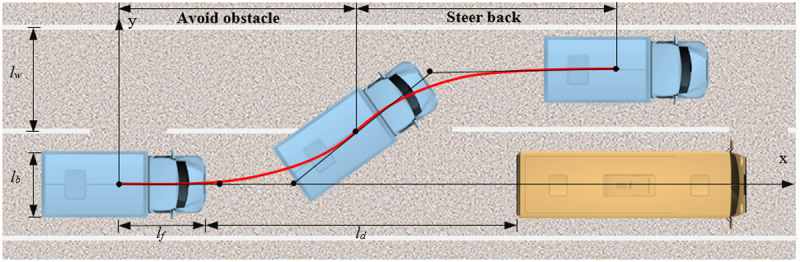
Trajectory planning of truck collision avoidance phase. The lane-changing process is divided into an obstacle avoidance phase and a trajectory recovery phase. The obstacle vehicle maintains a low, constant speed, and it is assumed that the subject truck has zero initial lateral velocity and lateral acceleration prior to lane change.

Leveraging findings that segmented trajectory optimization reduces computation time and enhances dynamic adaptability while isolating local disturbances [32], our methodology employs a segmented architecture (Fig 2), dividing the vehicle path into obstacle avoidance and lane-return phases based on roadway boundary constraints. The avoidance phase prioritizes spatial geometric relationships between the truck and obstacles to guarantee collision-free lane transitions, while the return phase optimizes for lane-change efficiency and ride comfort metrics specific to Tibet-bound heavy trucks. This dual-phase approach facilitates dynamic refinement of trajectory parameters. The complete solution utilizes a two-stage, three-dimensional Quartic Bézier curve formulation, demonstrating effective global optimization of trajectory planning objectives for high-altitude truck operations.

The complete lane-changing maneuver can be mathematically represented by the following parametric equations:


$$\begin{array}{c} \left\{ \begin{array}{c} @lx_{i}(t)=x_{i0}(1-t{)}^{4}+4x_{i1}(1-t{)}^{3}t+6x_{i2}(1-t{)}^{2}t^{2}+4x_{i3}(1-t)t^{3}+x_{i4}t^{4}\\ y_{i}(t)=y_{i0}(1-t{)}^{4}+4y_{i1}(1-t{)}^{3}t+6y_{i2}(1-t{)}^{2}t^{2}+4y_{i3}(1-t)t^{3}+y_{i4}t^{4} \end{array}\right.,\;t\in \left[0,1\right] \end{array}$$ (1)

Where i=1 models the avoidance phase and i=2 the return phase. The curvature $\kappa_{i}(t)$ at any curve point is expressed as:


$$\begin{array}{c} \kappa_{i}(t)=\frac{{\dot{x}}_{i}(t){\ddot{y}}_{i}(t)-{\ddot{x}}_{i}(t){\dot{y}}_{i}(t)}{\left({\dot{x}}_{i}^{2}(t)+{\dot{y}}_{i}^{2}(t\right){)}^{\frac{3}{2}}} \end{array}$$ (2)

The instantaneous curvature $\kappa_{i}(t)$ at any parametric point u along the Bézier curve can be expressed in terms of the vehicle’s kinematic state variables: where ${\dot{x}}_{i}(t)$ denotes longitudinal velocity, ${\dot{y}}_{i}(t)$ represents lateral velocity, ${\ddot{x}}_{i}(t)$ is longitudinal acceleration, and ${\ddot{y}}_{i}(t)$ corresponds to lateral acceleration. Differentiating Eq. (1) with respect to the path parameter *t* yields:


$$\begin{array}{c} \left\{ \begin{array}{c} @l{\dot{x}}_{i}(t)=\text{4}(x_{i\text{0}}-\text{4}x_{i\text{1}}+\text{6}x_{i\text{2}}-\text{4}x_{i\text{3}}+x_{i\text{4}})t^{\text{3}}+\text{1}\text{2}(-x_{i\text{0}}+\text{3}x_{i\text{1}}-\text{3}x_{i\text{2}}+x_{i\text{3}})t^{\text{2}}+\text{1}\text{2}(x_{i\text{0}}-\text{2}x_{i\text{1}}+x_{i\text{2}})t+\text{4}(-x_{i\text{0}}+x_{i\text{1}})\\ {\dot{y}}_{i}(t)=\text{4}(y_{i\text{0}}-\text{4}y_{i\text{1}}+\text{6}y_{i\text{2}}-\text{4}y_{i\text{3}}+y_{i\text{4}})t^{\text{3}}+\text{1}\text{2}(-y_{i\text{0}}+\text{3}y_{i\text{1}}-\text{3}y_{i\text{2}}+y_{i\text{3}})t^{\text{2}}+\text{1}\text{2}(y_{i\text{0}}-\text{2}y_{i\text{1}}+y_{i\text{2}})t+\text{4}(-y_{i\text{0}}+y_{i\text{1}}) \end{array}\right.\; \end{array}$$ (3)

By computing the second-order derivative of Eq. (1) with respect to the path parameter *t*, we obtain the curvature derivative relationship:


$$\begin{array}{c} \left\{ \begin{array}{c} @l{\ddot{x}}_{i}(t)=\text{1}\text{2}(x_{i\text{0}}-\text{4}x_{i\text{1}}+\text{6}x_{i\text{2}}-\text{4}x_{i\text{3}}+x_{i\text{4}})t^{\text{2}}+\text{2}\text{4}(-x_{i\text{0}}+\text{3}x_{i\text{1}}-\text{3}x_{i\text{2}}+x_{i\text{3}})t+\text{1}\text{2}(x_{i\text{0}}-\text{2}x_{i\text{1}}+x_{i\text{2}})\\ {\ddot{y}}_{i}(t)=\text{1}\text{2}(y_{i\text{0}}-\text{4}y_{i\text{1}}+\text{6}y_{i\text{2}}-\text{4}y_{i\text{3}}+y_{i\text{4}})t^{\text{2}}+\text{2}\text{4}(-y_{i\text{0}}+\text{3}y_{i\text{1}}-\text{3}y_{i\text{2}}+y_{i\text{3}})t+\text{1}\text{2}(y_{i\text{0}}-\text{2}y_{i\text{1}}+y_{i\text{2}}) \end{array}\right.\; \end{array}$$ (4)

### 2.2 Trajectory and velocity planning for obstacle avoidance phase

**Fig 3:**
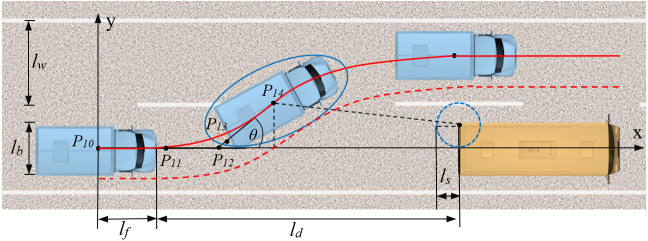
Collision avoidance trajectory planning. The lane-changing truck is enclosed by an elliptical boundary, where the red solid line represents its center of mass trajectory, and the red dashed line indicates the outermost contour trajectory of the truck’s envelope.

As shown in Fig 3, in the upper half of the obstacle avoidance trajectory curve, a unique quartic Bézier curve is determined by five control points, with coordinates $P_{10}(x_{10},y_{10})$, $P_{11}(x_{11},y_{11})$, $P_{12}(x_{12},y_{12})$, $P_{13}(x_{13},y_{13})$ and $P_{14}(x_{14},y_{14})$. At the vehicle’s initial position, the origin of the coordinate system coincides with the vehicle’s center of gravity, with the x-axis and y-axis representing the longitudinal and lateral driving directions, respectively. $l_{f}$ denotes the center-of-mass-to-front-bumper offset, $l_{b}$ is the vehicle width, $l_{d}$ is the distance between the front edge of the vehicle and the rear edge of the preceding vehicle in the current lane at the initial lane-changing position, $l_{s}$ is the reserved safety distance, and $l_{w}$ is the lane width.

In the upper half of the obstacle avoidance trajectory design, the vehicle is assumed to be traveling at a constant speed at the initial lane-changing position, with the longitudinal speed represented as $v_{x0}$. Prior to a lane-change command, we assume the vehicle travels straight with zero lateral speed and acceleration. This simplification aids controller design and stability analysis, although real-world vehicles might exhibit non-zero lateral dynamics. Therefore, the vehicle’s dynamic state at the initial position can be expressed as:


$$\begin{array}{c} \left\{ \begin{array}{c} @lx_{\text{1}}(\text{0})=x_{\text{1}\text{0}}=\text{0},{\dot{x}}_{\text{1}}(\text{0})=v_{x\text{0}},{\ddot{x}}_{\text{1}}(\text{0})=\text{0}\\ y_{\text{1}}(\text{0})=y_{\text{1}\text{0}}=\text{0},{\dot{y}}_{\text{1}}(\text{0})=\text{0},{\ddot{y}}_{\text{1}}(\text{0})=\text{0} \end{array}\right.\; \end{array}$$ (5)

Solving Equations (3), (4), and (5) yields the coordinates of the control points $P_{10}$, $P_{11}$ and $P_{12}$, as $P_{10}(0,0)$, $P_{11}(\frac{v_{x0}}{\text{4}},0)$ and $P_{12}(\frac{v_{x0}}{\text{2}},0)$. Thus, only the coordinates of control points $P_{13}$ and $P_{14}$ need to be determined to obtain the Bézier curve for the upper half of the obstacle avoidance trajectory. It is known that control point $P_{13}$ lies on the tangent of control point $P_{14}$. Considering the symmetry optimization of the trajectory offset and curvature of the Bézier curve, the coordinates of control points $P_{13}$ and $P_{14}$ satisfy:


$$\frac{y_{14}}{y_{13}}=\frac{x_{14}-x_{12}}{x_{13}-x_{12}}=\frac{x_{12}}{x_{12}-x_{11}}$$ (6)

The endpoint of the upper half lane-changing trajectory is at the lane boundary, so $y_{14}=\frac{l_{w}}{\text{2}}$. According to Eq. (6), $x_{13}=\frac{x_{14}}{\text{2}}+\frac{v_{x0}}{\text{4}}$, $y_{13}=\frac{l_{w}}{\text{4}}$. Therefore, only the x-coordinate of control point $P_{14}$ needs to be determined to obtain the corresponding upper half lane-changing trajectory.

Considering the large length, width, and turning radius of trucks entering Tibet, boundary constraints must be set for the lane-changing trajectory to ensure sufficient spacing between the vehicle and the obstacle ahead, preventing collisions. To meet the obstacle avoidance conditions, Fig 3 illustrates a feasible region for vehicle travel is defined around the planned Bézier curve trajectory. Following common engineering practice [33], we model the truck within the feasible region as an ellipse centered at the truck’s center of gravity $P_{14}$ and tangent to the feasible region boundary, with the semi-major axis being half the truck length plus the reserved safety distance and the semi-minor axis being half the truck width plus the reserved safety distance. The safety ellipse equation is:


$$\frac{{\left((x-x_{14})\cos\theta -(y-\frac{l_{w}}{\text{2}})\sin\theta \right)}^{2}}{{\left(l_{f}+l_{w}\right)}^{2}}+\frac{{\left((x-x_{14})\sin\theta +(y-\frac{l_{w}}{\text{2}})\cos\theta \right)}^{2}}{{\left(\frac{l_{b}}{\text{2}}+l_{w}\right)}^{2}}=1$$ (7)

Where, θ is the angle between the semi-major axis of the ellipse and the x-axis. Additionally, in scenarios where lane-changing is feasible for obstacle avoidance, the predominant type of frontal obstacles are predominantly slow-moving vehicles ahead.since the truck models entering Tibet are relatively concentrated, the width of the obstacle ahead is generally no greater than the width of the truck itself, so the width of the obstacle ahead is also selected as $l_{b}$. The closest point *N* between the preceding vehicle and the truck is selected as the center, with the reserved safety distance as the radius, forming the safety circle of the preceding vehicle. The center coordinates are $\left(l_{f}+l_{d},\frac{l_{b}}{\text{2}}\right)$, and the intersection point *N* between the line connecting the ellipse center and point *I* can be further determined. The distance between $P_{14}$ and *I* is:


$$d_{np}=\sqrt{\frac{{\left(l_{f}+l_{w}\right)}^{2}{\left(\frac{l_{b}}{\text{2}}+l_{w}\right)}^{2}({\left(l_{d}+l_{f}-x_{14}\right)}^{2}+{\left(\frac{l_{w}}{\text{2}}-\frac{l_{b}}{\text{2}}\right)}^{2})}{{\left(\frac{l_{b}}{\text{2}}+l_{w}\right)}^{2}{\left((l_{d}+l_{f}-x_{14})\cos\theta -(\frac{l_{w}}{\text{2}}-\frac{l_{b}}{\text{2}})\sin\theta \right)}^{2}+{\left(l_{f}+l_{w}\right)}^{2}{\left((l_{d}+l_{f}-x_{14})\sin\theta +(\frac{l_{w}}{\text{2}}-\frac{l_{b}}{\text{2}})\cos\theta \right)}^{2}}}$$ (8)

Then, based on geometric relationships, the conditions for vehicle obstacle avoidance planning are:


$$J_{11}(x_{14})=\frac{\text{1}}{l_{d}}\left(\sqrt{{\left(l_{d}+l_{f}-x_{14}\right)}^{2}+{\left(\frac{l_{w}}{\text{2}}-\frac{l_{b}}{\text{2}}\right)}^{2}}-d_{np}-l_{s}\right)>0$$ (9)

While ensuring obstacle avoidance, a larger reserved safety distance reduces the probability of collision with the obstacle ahead and improves lane-changing efficiency. However, with the y-coordinate of control point $P_{14}$ fixed, an excessively large reserved distance increases the trajectory curvature, indirectly increasing the risk of lateral instability. From the upper half of the obstacle avoidance trajectory curve, the curvature increases monotonically and reaches a maximum at control point $P_{14}$. Therefore, the curvature constraint for truck trajectory planning can be expressed as:


$$J_{12}(x_{14})=\frac{\kappa_{1}(t)}{\kappa_{\max}}\leq 1$$ (10)

Where, $\kappa_{\max}$ is the maximum curvature that satisfies the dynamic constraints of trucks entering Tibet. The optimization objective function is set as:


$$J_{1}(x_{14})=-w_{11}J_{11}(x_{14})+w_{12}J_{12}(x_{14})$$ (11)

Where, $w_{11}$ and $w_{12}$ are weight coefficients greater than 0. The obstacle avoidance trajectory planning problem in the upper half can then be transformed into a nonlinear optimization problem under constraints:


$$\begin{array}{c} \left\{ \begin{array}{c} @l\min J_{1}(x_{14})\\ J_{11}(x_{14})>0\\ J_{12}(x_{14})\leq 1 \end{array}\right. \end{array}$$ (12)

**Fig 4:**
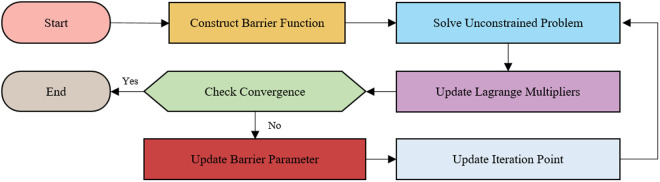
Interior-point method flowchart. The process terminates when convergence criteria are satisfied; otherwise, constraint parameters are updated and iterative solving proceeds.

For this nonlinear optimization problem, the interior-point method can be used to solve the objective function, as shown in the flowchart in Fig 4. After obtaining the x-coordinate $x_{14}$ of control point $P_{14}$, the coordinates of control point $P_{13}$ can be further determined, thus obtaining the corresponding Bézier curve and achieving the upper half lane-changing trajectory planning.

Based on the obstacle avoidance trajectory planning results, speed planning for the corresponding trajectory coordinates is performed, treating the real-time speed as the vertical coordinate of the truck trajectory, thereby establishing a real-time relationship between speed and truck position. Combining Eq. (1), the parametric equation for speed planning during vehicle travel can be expressed as:


$$v_{xi}(t)=z_{i}(t)=z_{i0}(1-t{)}^{4}+4z_{i1}(1-t{)}^{3}t+6z_{i2}(1-t{)}^{2}t^{2}+4z_{i3}(1-t)t^{3}+z_{i4}t^{4}$$ (13)

Taking the derivative of Eq. (13) yields the parametric equation for acceleration:


$$a_{xi}(t)={\dot{z}}_{i}(t)=4(z_{i0}-4z_{i1}+6z_{i2}-4z_{i3}+z_{i4})t^{3}+12(-z_{i0}+3z_{i1}-3z_{i2}+z_{i3})t^{2}+12(z_{i0}-2z_{i1}+z_{i2})t+4(-z_{i0}+z_{i1})$$ (14)

In the upper half of the obstacle avoidance trajectory, based on the speed and acceleration information at the truck’s initial position, we have:


$$\begin{array}{c} \left\{ \begin{array}{c} @lz_{1}(0)=z_{10}=v_{x0}\\ {\dot{z}}_{1}(0)=4(-z_{10}+z_{11})=0 \end{array}\right. \end{array}$$ (15)

From Eq. (15), $z_{10}=z_{11}=v_{x0}$. Thus, by determining the vertical coordinates $z_{12}$, $z_{13}$, $z_{14}$ of control points $P_{12}$, $P_{13}$ and $P_{14}$, the speed planning curve for the given trajectory can be obtained.

To ensure cargo safety and lateral stability during lane changes for Tibet-bound trucks, the lateral acceleration must not exceed $2.94m/s^{2}$ (0.3 times gravitational acceleration). preventing rollover caused by lane changes. Therefore, the constraint condition for longitudinal speed can be expressed as:


$$v_{x1}(t)=z_{1}(t)\leq \sqrt{2.94/\kappa_{1}(t)}$$ (16)

Compared to small vehicles, trucks entering Tibet have elevated centers of gravity, increasing the risk of rollover during turns and lane changes [34,35]. The quasi-static rollover threshold $T_{a}$ can be expressed as:


$$T_{a}=\frac{a_{y}}{9.8}=\frac{l_{a}}{2h_{g}[1+r_{\varphi }(1-h_{r}/h_{g})]}$$ (17)

Where, $l_{a}$ is the vehicle track width, $h_{g}$ is the truck’s center of gravity height, $r_{\varphi }$ is the truck’s roll rate, and $h_{r}$ is the roll center height. In actual driving, the dynamic rollover threshold is usually significantly lower than the quasi-static threshold, so a rollover adjustment coefficient $k_{\varphi }$ ($0<k_{\varphi }<1$) is selected, yielding the lateral acceleration threshold $9.8k_{\varphi }$ under these conditions. The calculation formula of $k_{\varphi }$ is shown in Equation (18). Table 4 lists the measured friction coefficients of typical sections of the highway leading to Tibet and the corresponding calculated values of the rollover reduction coefficients.

**Table 4:** Road friction and rollover thresholds for key Tibet highway segments. The table displays friction coefficients and corresponding rollover reduction coefficients under varying pavement conditions across four representative sections of the Qinghai-Tibet Highway.

Road Section	Pavement behavior	Dry		Damp/Waterlogged	
		μ	$k_{\varphi }$	μ	$k_{\varphi }$
Golmud-Kunlun Pass	Mild cracks, local repair	0.55 ~ 0.65	0.65-0.70	0.35 ~ 0.45	0.45 ~ 0.55
Wudaoliang-Tuotuo River	Reticular cracks and frost heave deformation	0.40 ~ 0.50	0.50 ~ 0.60	0.25 ~ 0.35	0.35 ~ 0.45
Tanggula Mountain Pass	Severe rutting and mud rolling	0.30 ~ 0.40	0.40 ~ 0.50	0.15 ~ 0.25	0.25 ~ 0.35
Dangxiong-Yangbajain	Exposed aggregates and pits	0.35 ~ 0.45	0.45 ~ 0.55	0.20 ~ 0.30	0.30 ~ 0.40


$$k_{\varphi }=\frac{2\mu h}{T}$$ (18)

Thus, the longitudinal speed constraint under rollover prevention is:


$$v_{x1}(t)=z_{1}(t)\leq \sqrt{9.8k_{\varphi }/\kappa_{1}(t)}$$ (19)

To prevent tire slip and ensure tire adhesion for heavy-duty trucks, the tire force should remain within the friction circle, with the corresponding constraint condition expressed as:


$$\sqrt{F_{x}^{2}+F_{y}^{2}}=\sqrt{{\left(m{\dot{z}}_{1}(t)\right)}^{2}+{\left(m\kappa_{1}(t)z_{1}^{2}(t)\right)}^{2}}\leq 9.8\mu m$$ (20)

Simplifying, we get:


$$\sqrt{{\left({\dot{z}}_{1}(t)\right)}^{2}+{\left(\kappa_{1}(t)z_{1}^{2}(t)\right)}^{2}}\leq 9.8\mu$$ (21)

Considering the impact of Tibet’s high-altitude, oxygen-deficient, and cold environment on vehicle power performance, the longitudinal acceleration should be limited [36], with the corresponding constraint condition expressed as:


$$\left|{\dot{z}}_{1}(t)\right|\leq \min(a_{x\max},9.8\left|\sin\theta \right|)$$ (22)

Here, θ denotes the road slope angle (positive for uphill), $a_{x\max}$ represents the maximum acceleration of the truck after reduction factors are applied to account for plateau environmental conditions, where $a_{x\max}$ is determined from measured data. Considering the critical influence of road grade on high-altitude maneuvers, the longitudinal acceleration constraint (Eq. 22) explicitly incorporates the slope angle θ, where positive θ denotes uphill conditions. Additionally, the planned speed should not exceed the maximum speed allowed by road conditions or the vehicle itself, with the corresponding constraint condition expressed as:


$$z_{1}(t)\leq v_{x\max}$$ (23)

Where, $v_{x\max}$ is the maximum speed limit. Under the above dynamic constraints, trucks entering Tibet need to complete emergency obstacle avoidance and lane changes as quickly as possible to avoid accidents or congestion caused by prolonged occupation of the opposite lane. With the trajectory planning results determined, the travel distance is fixed. Therefore, in the upper half speed planning, minimizing travel time is one of the optimization objectives, with the corresponding sub-objective function expressed as:


$$J_{21}(z_{12},z_{13},z_{14})=\frac{\text{1}}{\int_{0}^{1}z_{1}(t)dt}$$ (24)

Additionally, to reduce longitudinal and lateral impacts on trucks entering Tibet and improve driving smoothness and stability, the sub-objective functions for constraining longitudinal and lateral acceleration are expressed as:


$$\begin{array}{c} \left\{ \begin{array}{c} @lJ_{22}(z_{12},z_{13},z_{14})=\int_{0}^{1}\frac{\kappa_{1}(t)z_{1}^{2}(t)}{9.8\mu }dt\\ J_{23}(z_{12},z_{13},z_{14})=\int_{0}^{1}\frac{{\dot{z}}_{1}^{2}(t)}{a_{x\max}}dt \end{array}\right. \end{array}$$ (25)

The overall objective function can be expressed as:


$$J_{2}(z_{12},z_{13},z_{14})=w_{21}J_{21}+w_{22}J_{22}+w_{23}J_{23}$$ (26)

Where, $w_{21}$, $w_{22}$ and $w_{23}$ are weight coefficients greater than *0*. The speed planning problem in the upper half can then be transformed into a nonlinear optimization problem under constraints:


$$\begin{array}{c} \left\{ \begin{array}{c} @l\min J_{2}(z_{12},z_{13},z_{14})\\ z_{1}(t)\leq \sqrt{2.94/\kappa_{1}(t)}\\ z_{1}(t)\leq \sqrt{9.8k_{\varphi }/\kappa_{1}(t)}\\ \sqrt{{\left({\dot{z}}_{1}(t)\right)}^{2}+{\left(\kappa_{1}(t)z_{1}^{2}(t)\right)}^{2}}\leq 9.8\mu \\ \left|{\dot{z}}_{1}(t)\right|\leq a_{x\max}\\ z_{1}(t)\leq v_{x\max} \end{array}\right. \end{array}$$ (27)

Using the sequential quadratic optimization algorithm to solve this objective function yields the required $z_{12},z_{13},z_{14}$, thus determining the vertical and horizontal coordinates of all control points and achieving speed planning for the corresponding travel trajectory.

### 2.3 Trajectory and velocity planning for the lane-recovery phase

**Fig 5:**
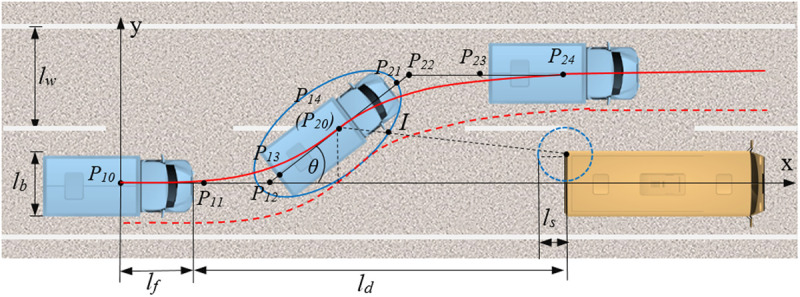
Truck trajectory planning of steering back. The trajectory endpoint of the collision avoidance phase serves as the starting point of the trajectory recovery phase, with control points $P_{22}$, $P_{23}$, and $P_{24}$ being collinear and satisfying tangent continuity conditions.

Based on the upper half obstacle avoidance trajectory planning, the endpoint of the upper half trajectory planning is used as the starting point for the lower half trajectory planning. Similarly, a quartic Bézier curve plans the lower half of the truck’s lane-change trajectory, guiding the truck from the road divider back to the lane centerline (Fig 5).

Fig 5 demonstrates that control points $P_{22}$, $P_{23}$ and $P_{24}$ lie on the same tangent line, so $y_{22}=y_{23}=y_{24}=l_{w}$. Additionally, control point $P_{20}$ coincides with $P_{14}$, and control points $P_{12}$, $P_{13}$, $P_{14}$, $P_{21}$ and $P_{22}$ lie on the same tangent line, with the angle between this tangent line and the x-axis represented as θ. Based on the geometric relationships in the figure, we have:


$$\frac{l_{w}}{x_{22}-x_{12}}=\frac{l_{w}/2}{x_{14}-x_{12}}=\tan\theta$$ (28)

From Eq. (3), $x_{22}=2x_{14}-\frac{v_{x0}}{\text{2}}$. Thus, the coordinates of control points $P_{20}$ and $P_{22}$ in the xy-plane can be expressed as $\left(x_{14},\frac{l_{w}}{\text{2}}\right)$ and $\left(2x_{14}-\frac{v_{x0}}{\text{2}},l_{w}\right)$, respectively. Similarly, the coordinate relationship of control point $P_{21}$ satisfies:


$$\frac{y_{21}}{x_{21}-x_{12}}=\tan\theta$$ (29)

Thus, $y_{21}=\tan\theta (x_{21}-\frac{v_{x0}}{\text{2}})$ can be solved. Additionally, consistent with the proportional relationship expressed in Eq. (6), the proportional relationship in the lower half Bézier curve can also be expressed as:


$$\frac{P_{23}P_{24}}{P_{22}P_{24}}=\frac{x_{23}-x_{22}}{x_{24}-x_{22}}=\frac{P_{21}P_{22}}{P_{20}P_{22}}=\frac{x_{22}-x_{21}}{l_{w}/2\tan\theta }$$ (30)

Thus, $\begin{array}{c} x_{24}=\frac{(x_{23}-x_{22})l_{w}}{2(x_{22}-x_{21})\tan\theta }+x_{22} \end{array}$.

Combining Eq. (29) and Eq. (30), only the x-coordinates of control points $P_{21}$ and $P_{23}$ need to be determined to obtain all control point coordinates, thus determining the unique lower half lane-changing trajectory curve.

In the lower half return trajectory planning, the real-time speed is also treated as the vertical coordinate of the vehicle trajectory. Control point $P_{20}$ coincides with control point $P_{14}$. Considering the continuity of vehicle speed and acceleration, the speed and acceleration at control point $P_{20}$ should be consistent with those at control point $P_{14}$. Therefore, the speed and acceleration information at the initial position of the return trajectory can be expressed as:


$$\begin{array}{c} \left\{ \begin{array}{c} @lz_{2}(0)=z_{20}=z_{1}(1)\\ {\dot{z}}_{2}(0)=4(-z_{20}+z_{21})={\dot{z}}_{1}(1) \end{array}\right. \end{array}$$ (31)

$$\frac{x_{22}-x_{20}}{x_{20}-x_{12}}=\frac{z_{22}-z_{20}}{z_{20}-z_{12}}$$ (32)

Calculating $z_{21}=z_{1}(1)+\frac{{\dot{z}}_{1}(1)}{\text{4}}$, $z_{22}=2z_{1}(1)-z_{12}$. Similarly, by analogy with the proportional relationship in Eq. (6), we have:


$$\frac{z_{22}-z_{21}}{z_{20}-z_{20}}=\frac{z_{23}-z_{22}}{z_{24}-z_{22}}$$ (33)

Solving Eq. (33) yields:


$$z_{23}=\left(1-\frac{{\dot{z}}_{1}(1)}{4(z_{1}(1)-z_{12})}\right)z_{24}-2(z_{1}(1)-z_{12})+\frac{{\dot{z}}_{1}(1)(2(z_{1}(1)-z_{12}))}{4(z_{1}(1)-z_{12})}$$ (34)

Thus, only the speed $z_{24}$ at the endpoint of the return trajectory needs to be determined to obtain the speed $z_{23}$ at control point $P_{23}$, thereby obtaining the overall speed planning curve.

In the upper half obstacle avoidance trajectory planning, the primary goal was obstacle avoidance, and vehicle trajectory planning was performed considering road curvature constraints, followed by speed planning based on the trajectory planning results. Since the vehicle has already achieved obstacle avoidance in the upper half, the goal of trajectory planning in the return process is to return the vehicle to the lane as quickly as possible within the allowed range while considering road curvature constraints, thereby reducing lane-changing distance and improving lane-changing efficiency. Additionally, speed planning results directly affect lane-changing efficiency, so the return process trajectory planning and speed planning problems need to be combined for optimal design. [5,8,12]

Combining the integrated design objectives of trajectory planning and speed planning in the return process, the optimization objective function is set as:


$$J_{3}(x_{21},x_{23},z_{24})=w_{31}J_{31}(x_{21},x_{23},z_{24})+w_{32}J_{32}(x_{21},x_{23},z_{24})$$ (35)

Where, $w_{31}$ and $w_{32}$ are weight coefficients greater than 0. The sub-objective function $J_{31}$ can be expressed as:


$$J_{31}(x_{21},x_{23},z_{24})=x_{24}+\int_{0}^{1}z_{2}(t)dt$$ (36)

The sub-objective function $J_{31}$ is directly related to vehicle trajectory planning and speed planning requirements, characterizing the endpoint position of the lane change and the overall travel distance. Minimizing $J_{31}$ helps ensure quick lane changes while reducing the total length of the curved trajectory, avoiding additional travel risks, and improving lane-changing efficiency while reducing energy loss. The optimization results of $J_{31}$ significantly affect the curvature of the vehicle’s travel trajectory. Therefore, configuring a larger curvature within an appropriate range during the return process helps achieve quick lane changes, but excessively large curvature also increases the risk of lateral instability. Thus, the curvature constraint is set as $J_{21}=K_{2}(0)\leq K_{\max}$. The sub-objective function $J_{32}$ also affects speed planning results. Since $J_{31}$ can constrain trajectory planning and speed planning results through the lane change endpoint and travel distance, the design of $J_{32}$ considers the impact of longitudinal and lateral acceleration on vehicle stability and smoothness. The sub-objective function $J_{32}$ can be expressed as:


$$J_{32}(x_{21},x_{23},z_{24})=J_{321}+J_{322}$$ (37)

Where, $J_{321}(x_{21},x_{23},z_{24})=\int_{0}^{1}\frac{\kappa_{1}(t)z_{2}^{2}(t)}{\mu g}dt$, $J_{322}(x_{21},x_{23},z_{24})=\int_{0}^{1}\frac{{\dot{z}}_{2}^{2}(t)}{a_{x\max}}dt$. Finally, considering the dynamic constraints involved in speed planning in the obstacle avoidance trajectory, the trajectory planning and speed planning problems in the return process can be integrated into the following nonlinear optimization problem with constraints:


$$\begin{array}{c} \left\{ \begin{array}{c} @l\min J_{3}(x_{21},x_{23},z_{24})\\ \kappa_{2}(0)\leq \kappa_{\max}\\ z_{2}(t)\leq \sqrt{2.94/\kappa_{2}(t)}\\ z_{2}(t)\leq \sqrt{9.8k_{\varphi }/\kappa_{2}(t)}\\ \sqrt{{\left({\dot{z}}_{2}(t)\right)}^{2}+{\left(\kappa_{2}(t)z_{2}^{2}(t)\right)}^{2}}\leq 9.8\mu \\ \left|{\dot{z}}_{2}(t)\right|\leq a_{x\max}\\ z_{2}(t)\leq v_{x\max} \end{array}\right. \end{array}$$ (38)

Using the sequential quadratic optimization algorithm to solve this objective function yields the required $x_{21}$, $x_{23}$, and $z_{24}$, thus determining the vertical and horizontal coordinates of all control points and completing trajectory planning and speed planning for the return process.

## 3. Results and discussion

We conducted numerical simulations in Python (Spyder 4.2.5). Trajectory and velocity optimization for obstacle avoidance and recovery phases were solved separately using parameters in Table 5.

**Table 5:** Simulation parameters. The simulation parameters include vehicle parameters, speed parameters, road parameters, and weight parameters.

Parameters	Initial configuration
Vehicle Parameters	Center-of-mass-to-front-bumper offset, $l_{f}=9$ Vehicle width, $l_{b}=2.5$ Reserved safety distance, $l_{s}=1$ Lane width, $l_{w}=3$ Rollover adjustment coefficient, $k_{\varphi }=0.5$
Speed Parameters	Initial speed, $v_{x0}=4,6,8,10,12,14,16$ Maximum speed limit, $v_{\max}=16.7$ Maximum acceleration, $a_{x\max}=0.147$
Road Parameters	Distance between the front edge of the vehicle and the rear edge of the preceding vehicle, $l_{d}=10,15,20,25,30,35,40$ Coefficient of friction, μ=0.4 Maximum curvature, $\kappa_{\max}=1/125$
Coefficient of weight	$w_{11}=0.5$, $w_{12}=0.5$ $w_{21}=1$, $w_{22}=1$, $w_{23}=1$ $w_{31}=1$, $w_{32}=1$

**Fig 6:**
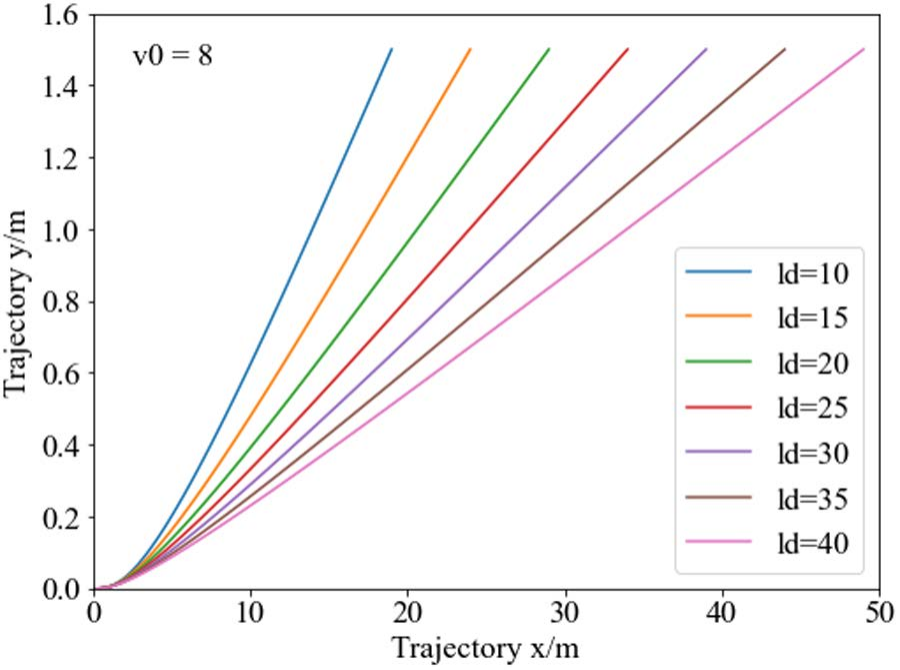
Obstacle avoidance trajectories at varying obstacle spacing. At an initial speed of 8 m/s, as the obstacle spacing increases, the lateral coordinate of the trajectory endpoint during the collision avoidance phase increases accordingly.

From Fig 6, the analysis shows that at a constant initial speed, the x-coordinates of the planned obstacle avoidance trajectory points vary with obstacle spacing. The larger the allowed lane-changing distance, the larger the x-coordinate of the obstacle avoidance trajectory endpoint, and the smaller the curvature of the obstacle avoidance trajectory. This indicates that with sufficient obstacle spacing, the planned vehicle obstacle avoidance trajectory is relatively smooth, effectively ensuring longitudinal and lateral stability.

**Fig 7:**
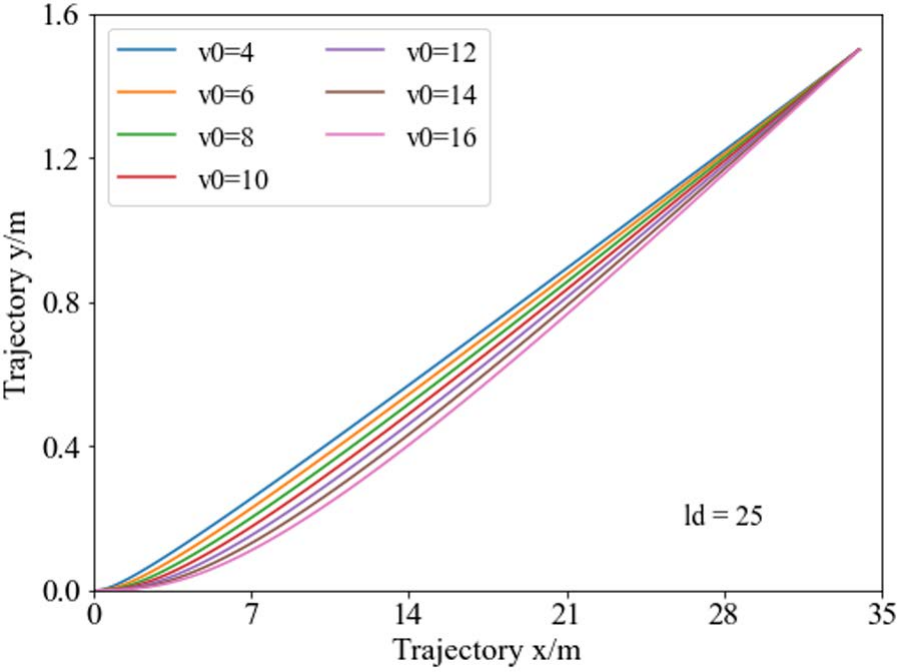
Collision avoidance trajectory curves at varying initial vehicle speeds. When the obstacle spacing is fixed, the trajectory endpoint position during the collision avoidance phase remains unchanged across different initial speeds.

Fig 7 demonstrates that with the lane-changing distance set to 25 meters, the vehicle obstacle avoidance trajectory planning curves under different initial speeds are shown. Analysis reveals, with a fixed obstacle spacing, the lane-changing trajectory endpoints are the same under different speeds. Additionally, with higher initial speeds, the curvature at the trajectory endpoint is relatively larger.

**Fig 8:**
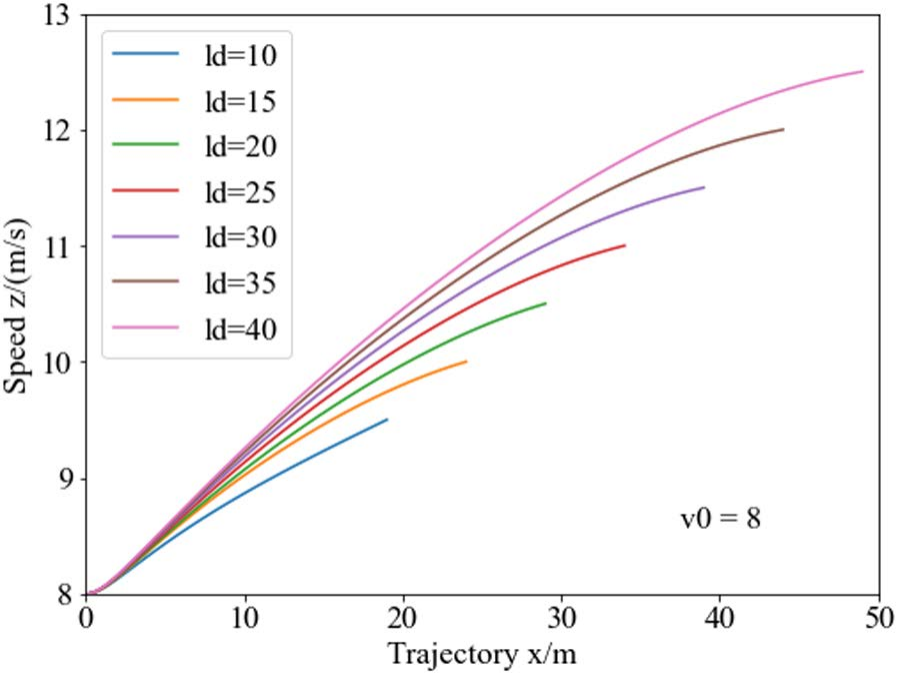
Speed planning curves for collision avoidance under varying obstacle spacing. As the obstacle spacing increases, the vehicle’s terminal speed during the collision avoidance phase rises, with the speed differential between different obstacle spacing progressively amplifying as the avoidance maneuver progresses.

Fig 8 illustrates the speed planning curves under different obstacle spacing conditions with an initial speed of 8 m/s. Analysis reveals, in the initial stage of lane changing, the speed planning results are similar, but after about 5 meters, the speeds diverge significantly. Owing to different lane-changing endpoints under different obstacle spacing conditions, the speeds at the endpoints differ noticeably, with achieving a maximum velocity of 12.5 m/s (45 km/h) at the endpoint for a lane-changing distance of 40 meters.

**Fig 9:**
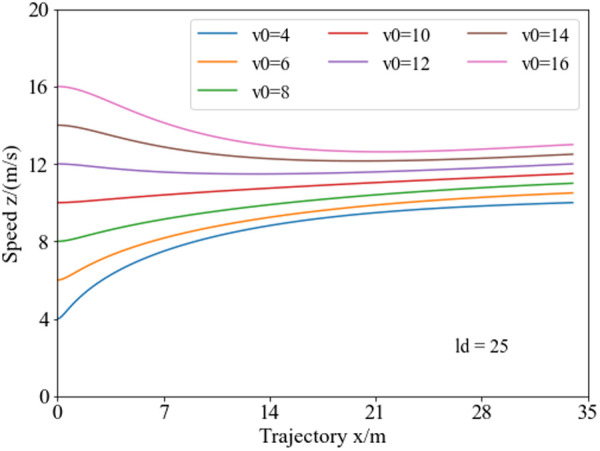
Collision avoidance speed planning curves for varying initial vehicle speeds. When the initial speed is relatively low, the vehicle accelerates to the endpoint of the collision avoidance phase within a fixed distance. Conversely, at higher initial speeds, it decelerates to reach the endpoint. Regardless of whether accelerating or decelerating, the magnitude of acceleration progressively diminishes as the collision avoidance maneuver develops.

Fig 9 illustrates the speed planning curves under different initial speeds with a lane-changing distance of 25 meters. Analysis reveals, with lower initial speeds, the vehicle accelerates more significantly to reach the endpoint within a fixed travel distance, improving lane-changing efficiency. As the initial speed increases, the acceleration during obstacle avoidance gradually decreases. When the initial speed exceeds 10 m/s, the vehicle decelerates to a certain speed to meet stability requirements and then travels steadily.

**Fig 10:**
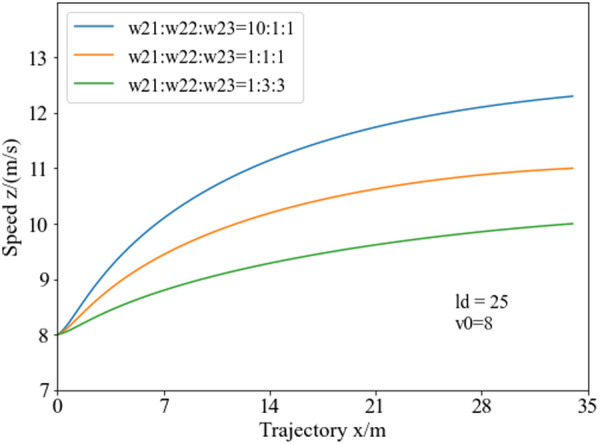
Collision avoidance speed planning curves under varying weight coefficients. When a higher $w_{21}$ weighting is assigned to the acceleration parameter, the vehicle accelerates more rapidly and reaches the planned endpoint at a higher terminal speed. Conversely, when greater $w_{22}$ and $w_{23}$ weightings are allocated to the steering and stability parameters, the vehicle accelerates more gradually and attains the same planned endpoint at a relatively lower speed.

In speed planning, the ratio of weights for different objective functions in Eq. (24) also affects the speed planning results. Fig 10 illustrates the speed planning curves under different weight coefficients, with an initial speed of 8 m/s and a lane-changing distance of 25 meters. Analysis reveals, when the weight coefficient ratio for the speed planning objective function is 10:1:1, the objective function tends to improve lane-changing efficiency during obstacle avoidance. Therefore, with a fixed lane-changing endpoint, the vehicle accelerates more significantly, resulting in higher overall speed during lane changing and reduced lane-changing time. When the weight coefficient ratio is 1:1:1, the vehicle considers both lane-changing efficiency and driving smoothness and comfort, resulting in more uniform acceleration during lane changing and effectively reducing vehicle roll and yaw changes during turns. When the weight coefficient ratio is 1:3:3, the speed planning curve’s curvature changes more uniformly, with the vehicle approaching uniform acceleration, and the overall acceleration and speed at the lane-changing endpoint are relatively lower, indicating a greater focus on vehicle stability performance.

**Fig 11:**
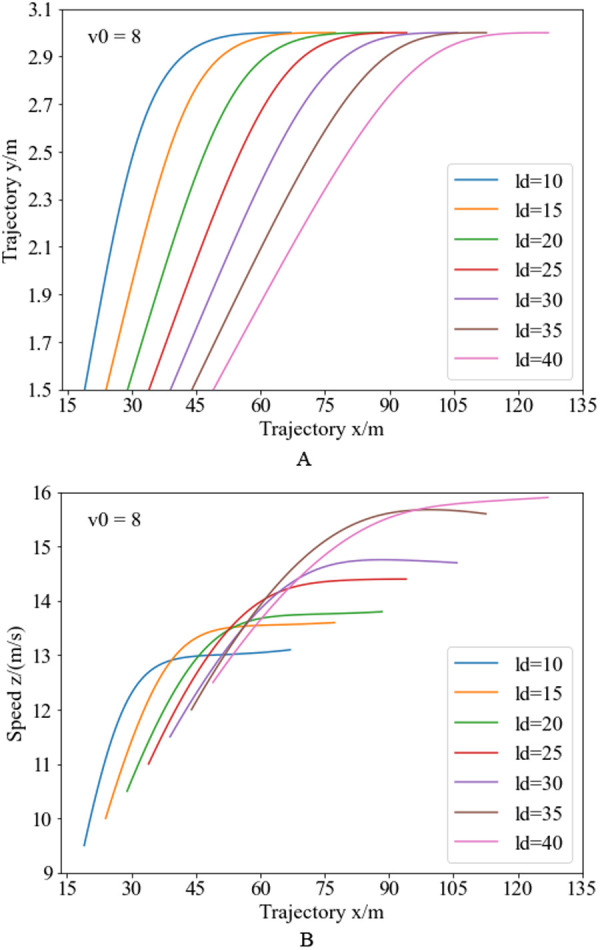
Return trajectory and speed planning curve under varying obstacle spacing. Figure (a) presents the trajectory planning results, while Figure (b) displays the speed planning results.

Based on the trajectory planning and speed planning design methods for the return process, the return trajectory is designed based on the obstacle avoidance trajectory, with all weight coefficients for the objective functions set to 1. Combining the vehicle position and speed at the lane-changing endpoint in Figs 6 and 8, the return trajectory and speed planning results under different obstacle spacing conditions are shown in Fig 11. Fig 11a illustrates the lower half return trajectory planning results under different obstacle spacing conditions. Analysis reveals, with larger obstacle spacing, the initial position coordinates of the lower half trajectory are larger, and the initial curvature is smaller, resulting in a longer travel distance for the return trajectory. Additionally, since lane-changing efficiency is considered in the return trajectory design, with larger obstacle spacing, the initial curvature is relatively smaller, and the overall lane-changing distance is relatively larger. Therefore, with larger obstacle spacing, the optimization results show a slower reduction rate in trajectory curvature, allowing for some trajectory correction to improve lane-changing efficiency. Fig 11b illustrates the speed planning results for the return trajectory under different obstacle spacing conditions. From the speed planning results for the upper half obstacle avoidance trajectory under different obstacle spacing conditions, Analysis reveals the endpoint speeds differ under different obstacle spacing conditions. Therefore, the initial speeds at the starting position of the return trajectory also differ. With smaller obstacle spacing, the initial speed and return endpoint position coordinates are relatively smaller, and to improve lane-changing efficiency, the speed increases more rapidly, with the vehicle approaching constant speed near the lane-changing endpoint. With larger obstacle spacing, the initial speed and return endpoint position coordinates are relatively larger, and the speed increases more slowly, ensuring lane-changing efficiency while considering vehicle stability, comfort, and smoothness.

**Fig 12:**
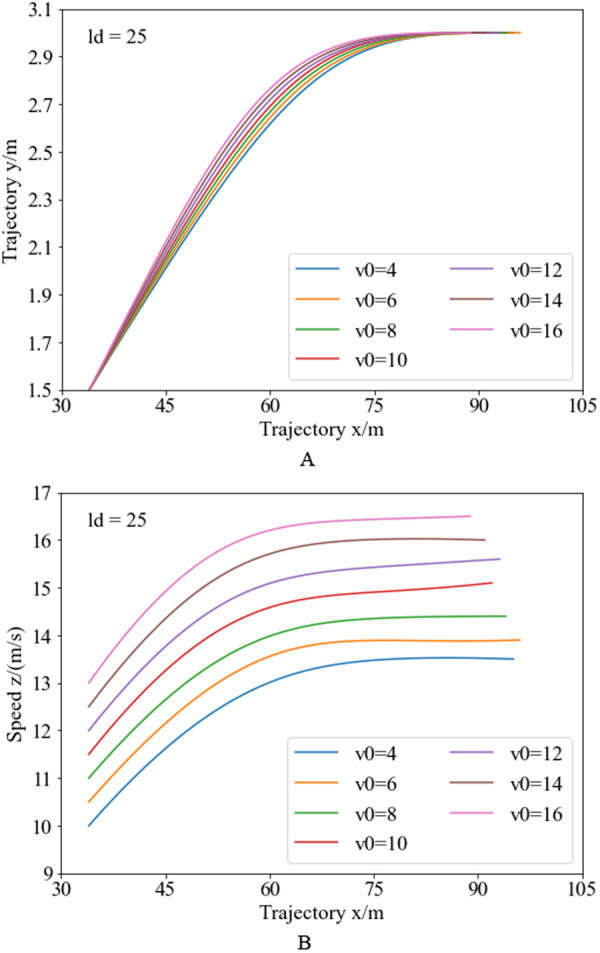
Return trajectory and speed planning curve for varying initial vehicle speeds. Figure (a) presents the trajectory planning outcomes for the steering recovery phase, while Figure (b) displays the speed planning outcomes during the same phase.

Combining the vehicle position and speed at the lane-changing endpoint in Figs 7 and 9, the return trajectory and speed planning results under different initial speeds are shown in Fig 12. Fig 12a illustrates the return trajectory planning results under different initial speeds. In the obstacle avoidance trajectory, with the same obstacle spacing but different initial speeds, the lane-changing trajectory endpoints are the same, so the starting positions of the return trajectory are also the same. With lower initial speeds, the initial curvature of the return trajectory is relatively smaller, and the curvature reduction rate is relatively slower, resulting in a larger endpoint position coordinate for the return trajectory under low-speed conditions. With higher initial speeds, the initial curvature of the return trajectory is relatively larger, and the curvature change rate is relatively faster, allowing the vehicle to complete the lane change in a shorter distance, with relatively higher lane-changing efficiency. Fig 12b illustrates the speed planning results for the return trajectory under the same obstacle spacing but different initial speeds. Analysis reveals during the return trajectory, without the influence of obstacles, and under the premise of ensuring vehicle stability and dynamic constraints, more indicators in the trajectory planning objective function are used to improve lane-changing efficiency. Additionally, since the primary goal of the return trajectory is to improve lane-changing efficiency, under high-speed conditions, the vehicle can complete the lane change more quickly and with a shorter travel distance, while under low initial speed conditions, owing to dynamic constraints, the speed cannot increase rapidly, resulting in a longer travel distance to complete the lane change.

**Fig 13:**
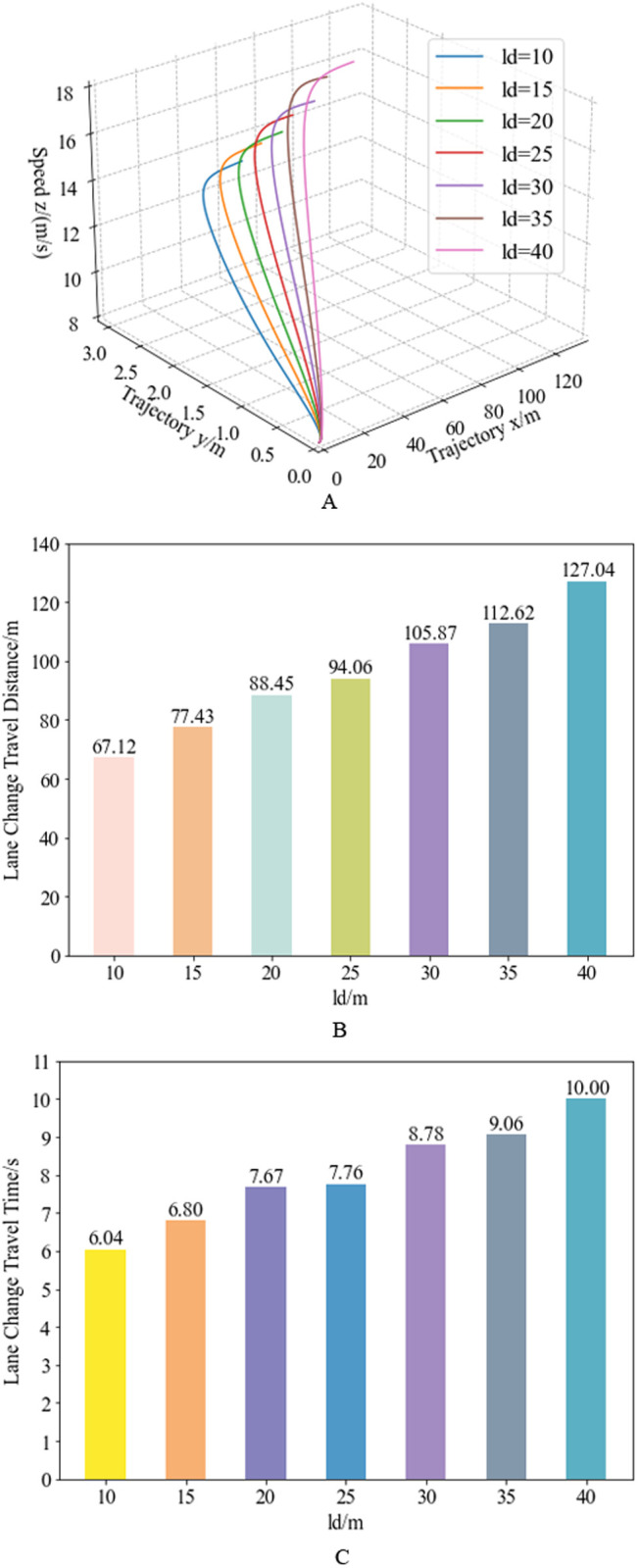
Results of 3-D Bézier trajectory planning with varying obstacle spacing. Figure (a) depicts the 3D curves of trajectory and speed planning under varying obstacle distances at an initial velocity of 8 m/s. Figure (b) presents the lane-change travel distance across different obstacle spacing, while Figure (c) displays the lane-change duration for corresponding obstacle distance conditions.

Combining the trajectory planning curves under different obstacle spacing conditions in Figs 6 and 11a, the complete quartic Bézier trajectory planning curve for the entire lane-changing process is obtained. Combining the speed planning curves under different obstacle spacing conditions in Figs 8 and 11b, the complete quartic Bézier speed planning curve for the entire lane-changing process is obtained. Integrating the trajectory planning and speed planning results, the three-dimensional, segmented quartic Bézier curve planning results under different obstacle spacing conditions are shown in Fig 13. Fig 13a illustrates the three-dimensional planning trajectory comparison under the same initial speed (10 m/s) but different obstacle spacing conditions. Fig 13b and 13c show the travel distance and travel time comparison for vehicle lane changes under different obstacle spacing conditions. Analysis reveals, with a fixed initial speed, the larger the actual obstacle spacing, the longer the travel distance in the planning results, and the longer the corresponding lane-changing travel time.

**Fig 14:**
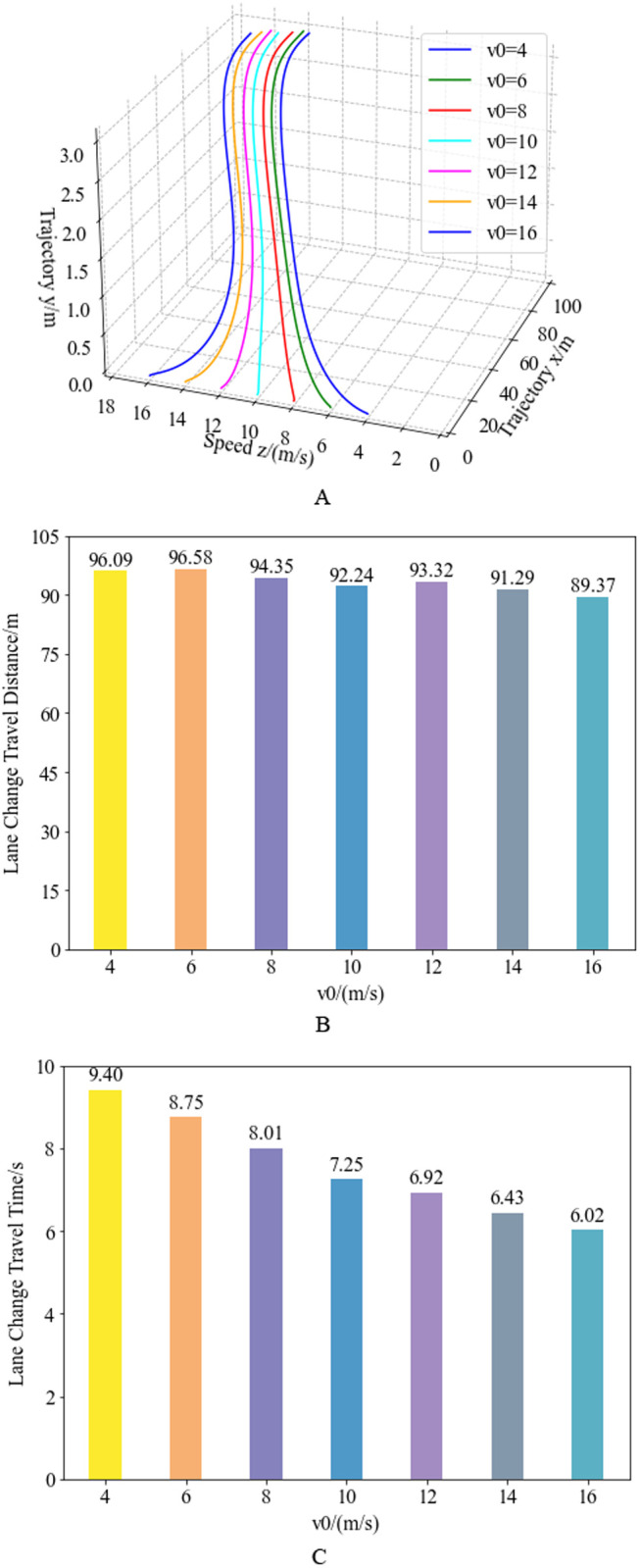
Results of 3D Bézier trajectory planning for varying initial vehicle speeds. Figure (a) depicts the 3D curves of trajectory and speed planning at varying initial velocities under a fixed obstacle distance of 25 m. Figure (b) presents the lane-change travel distance across different initial speeds, while Figure (c) displays the lane-change duration for corresponding initial velocity conditions.

Similarly, combining the trajectory planning curves under different initial speeds in Figs 7 and 12a, the complete quartic Bézier curve for the entire lane-changing process is obtained. Combining the speed planning curves under different initial speeds in Figs 9 and 12b, the complete quartic Bézier speed planning curve for the entire lane-changing process is obtained. Integrating the trajectory planning and speed planning results, the three-dimensional, segmented quartic Bézier curve planning results under different initial speeds are shown in Fig 14. Fig 14a illustrates the three-dimensional planning trajectory comparison under the same obstacle spacing (25 meters) but different initial speeds. Figs 14b and 14c show the travel distance and travel time comparison for vehicle lane changes under different initial speeds. Analysis reveals, with a fixed obstacle spacing, the higher the initial speed of the truck, the shorter the travel distance in the planning results, and the shorter the corresponding lane-changing travel time.

**Fig 15:**
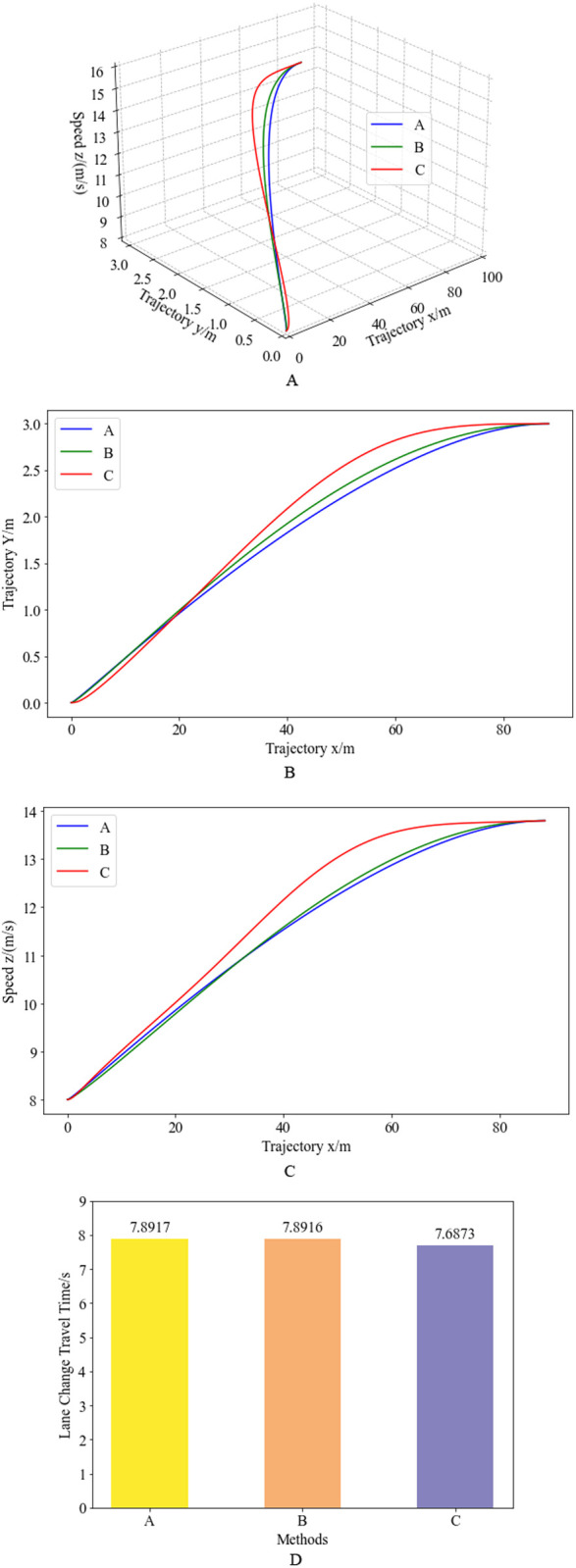
Trajectory planning results of different planning algorithms under fixed driving mileage. Figure (a) depicts the 3D trajectory and speed planning curves for three distinct methods. Figure (b) presents the 2D trajectory projections of these methods, while Figure (c) illustrates the speed profiles as functions of longitudinal displacement for each approach. Figure (d) compares the lane-change duration across all three methods under a representative scenario with initial velocity 8 m/s and obstacle spacing 25 m.

To further demonstrate the advantages of the proposed three-dimensional, segmented quartic Bézier curve-based vehicle trajectory planning method, a comparison with quartic and cubic Bézier curves is conducted. The initial speed is set to 8 m/s, and the obstacle spacing is set to 25 meters for the simulation. From the planning results, the lane-changing travel distance based on the three-dimensional, segmented quartic Bézier curve is 94.07 meters. Therefore, a fixed travel distance of 94.07 meters is used for comparison with different planning algorithms. The trajectory planning comparison results under the fixed travel distance are shown in Fig 15, where A, B, and C represent the results based on the cubic Bézier curve, the quartic Bézier curve, and the proposed method, respectively. Fig 15 demonstrates that under the same initial speed, obstacle spacing, and travel distance, the trajectory planning results of the three methods are relatively similar. The proposed method’s lane-changing curve is smoother overall, while the trajectories based solely on the quartic and cubic Bézier curves have relatively larger curvature in the initial obstacle avoidance stage. The speed planning results of the three methods differ significantly. Since the proposed method’s trajectory has relatively smaller curvature in the initial obstacle avoidance stage, the overall speed planning tends to accelerate more significantly to improve lane-changing efficiency and maneuverability. In the later stage with lower curvature, the speed is significantly higher than the other two methods, also improving lane-changing efficiency and maneuverability. The overall lane-changing travel time comparison further proves that the proposed method effectively achieves a 2.59% reduction in lane-change duration while ensuring all stability constraints.

Several assumptions limit this study’s scope. First, we assumed zero initial lateral velocity and acceleration (Eq. 5) to decouple longitudinal-lateral dynamics during Bézier curve derivation. This simplification enables tractable optimization but may not hold under transient lateral dynamics. Second, although Tibet’s mountainous terrain introduces significant road inclination, our 3D Bézier formulation did not explicitly parameterize grade. We prioritized dominant lateral stability challenges (curvature-induced rollover, narrow lanes), incorporating grade effects only via longitudinal acceleration limits in dynamic constraints (Eq. 22). Third, we assumed constant tire-road friction (Eq. 21), overlooking dynamic variations common on Tibetan highways (e.g., ice, gravel). These simplifications inherently restrict generalizability to inclined roads or low-friction scenarios.

Future work will address these limitations:

- Integrating road inclination: Incorporate Digital Elevation Models (DEMs) and powertrain-grade coupling into the Bézier parameterization, particularly for asymmetric upgrade/downgrade kinematics.
- Enhancing robustness: Develop stochastic lateral dynamic models with non-zero initial velocities and road disturbances.
- Large-scale validation: Conduct field tests across Tibet’s major corridors (e.g., G318, S307) using diverse freight fleets, quantifying performance under real-world noise, mixed traffic, and variable adhesion.
- Beyond Tibet, this framework’s modular architecture—integrating altitude-specific constraints (power attenuation, rollover thresholds)—provides a transferable paradigm for autonomous logistics in extreme terrains (e.g., Andean mining, Arctic supply chains).
- Subsequent research will also explore V2X-enabled cooperative planning to resolve multi-vehicle conflicts in single lanes, potentially reducing emergency maneuver distances by 15–22% [25,30].

## 4. Conclusion

This work proposes a novel 3D segmented quartic Bézier curve framework for integrated trajectory and velocity planning, enhancing maneuverability and stability of plateau-operating heavy-duty trucks. Unlike decoupled approaches, our method integrates spatial coordinates (XY plane) and velocity profiles (Z-axis) into a unified parametric representation using G²-continuous quartic Bézier curves. This integration resolves the dynamic infeasibility inherent in decoupled planning. Compared to cubic Bézier curves [29], quartic curves achieve G² continuity (curvature continuity), eliminating sudden acceleration jumps at trajectory joints (Fig 15). The methodology incorporates altitude-specific dynamic constraints—including powertrain performance attenuation, rollover risk thresholds, and tire friction circle limitations—within a computationally efficient dual-phase optimization structure. This structure prioritizes collision avoidance during lane deviation while optimizing lane-return efficiency, achieving real-time trajectory synthesis through sensor fusion (vision/radar) and execution via drive-by-wire systems under Tibet’s operational constraints.

Validation demonstrates significant performance improvements: Trajectory curvature reduces by 12–18% at hairpin curves (Figs 6 and 15), and lane-change duration decreases by 2.59% (Fig 15c) while maintaining lateral stability. The framework combines mathematical guarantees (convex hull safety) with computational practicality, offering a robust approach for high-altitude autonomous navigation. This closed-loop perception-planning-execution architecture (Fig 1) addresses critical stability-efficiency trade-offs in specialized terrains. It provides a foundational methodology for autonomous freight systems in extreme topographies like the Tibetan Plateau.

## Supporting information

S1 File Data_ImagePoints. The raw data for all figures in the article.
(XLSX)
